# Earth Surface Deformation in the North China Plain Detected by Joint Analysis of GRACE and GPS Data

**DOI:** 10.3390/s141019861

**Published:** 2014-10-22

**Authors:** Renli Liu, Jiancheng Li, Hok Sum Fok, C.K. Shum, Zhao Li

**Affiliations:** 1 School of Water Resources and Hydropower Engineering, Wuhan University, 8 Donghu South Road, Wuhan 430072, China; E-Mail: lrlwuhan@gmail.com; 2 School of Geodesy and Geomatics, Wuhan University, 129 Luoyu Road, Wuhan 430079, China; E-Mail: xshhuo@sgg.whu.edu.cn; 3 School of Earth Sciences, Ohio State University, 275 Mendenhall Lab., 125 S. Oval Mall, Columbus, OH 43210, USA; E-Mail: ckshum@osu.edu; 4 Institute of Geodesy & Geophysics, Chinese Academy of Sciences, Wuhan 430077, China; 5 Faculté des Sciences, de la Technologieet de la Communication, University of Luxembourg, L-1359 Luxembourg; E-Mail: zhao.li@uni.lu

**Keywords:** GRACE, GPS, time-variable gravity field, loading, surface deformation

## Abstract

Mass redistribution of the Earth causes variable loading that deforms the solid Earth. While most recent studies using geodetic techniques focus on regions (such as the Amazon basin and the Nepal Himalayas) with large seasonal deformation amplitudes on the order of 1–4 cm due to hydrologic loading, few such studies have been conducted on the regions where the seasonal deformation amplitude is half as large. Here, we use joint GPS and GRACE data to investigate the vertical deformation due to hydrologic loading in the North China Plain, where significant groundwater depletion has been reported. We found that the GPS- and GRACE-derived secular trends and seasonal signals are in good agreement, with an uplift magnitude of 1–2 mm/year and a correlation of 85.0%–98.5%, respectively. This uplift rate is consistent with groundwater depletion rate estimated from GRACE data and *in-situ* groundwater measurements from earlier report studies; whereas the seasonal hydrologic variation reflects human behavior of groundwater pumping for agriculture irrigation in spring, leading to less water storage in summer than that in the winter season. However, less than 20% of weighted root-mean-squared (WRMS) reductions were detected for all the selected GPS stations when GRACE-derived seasonal deformations were removed from detrended GPS height time series. This discrepancy is probably because the GRACE-derived seasonal signals are large-scale, while the GPS-derived signals are local point measurements.

## 1. Introduction

The redistribution of atmospheric, oceanic, cryospheric and terrestrial hydrologic masses as well as their interactions cause variable loading, and thus, deform the solid Earth both horizontally and vertically [1,2]. This process is referred to as surface deformation (or displacement) due to surface loading, in which the vertical displacement is of primary interest. Among the abovementioned geophysical mass redistribution processes, the effect of regional scale terrestrial water variation is less known due to the spatially sparse *in-situ* measurements and the complexity of terrestrial hydrologic cycles [3,4].

With the advances in space geodetic sensors, such as satellite gravimetry and Global Positioning System (GPS), spatially-distributed data are increasingly available and have been used in the estimation of water storage at the basin, regional, and global scales [5–7]. The Gravity Recovery and Climate Experiment (GRACE) tandem satellite gravimetry mission, launched in 2002, provides a unique opportunity for studying the Earth's global temporal gravity variations, and hence, the temporal surface mass variation at monthly sampling and a spatial scale larger than 330 km. Over the land surface, GRACE time-variable gravity fields mainly reflect the hydrologic mass redistribution at seasonal timescales, which is useful for quantifying the temporal variation of total water storage changes [8]. It can also be used to infer Earth surface deformation [2]. The surface deformation due to loading caused by the redistribution of water mass can be observed by GPS [9]. These two geodetic techniques, *i.e.*, GRACE and GPS, complement each other in terms of spatial and temporal coverage, and hence, allow cross-validation of climate change-driven Earth surface deformation signals.

Several studies have been conducted in areas with large hydrologic signal using GRACE and GPS techniques. Davis *et al.* [10] found high correlation of annual hydrologic variations between GRACE- and GPS-derived vertical surface displacements from their respective residual height time series in the Amazon Basin. Consistent seasonal displacements between GPS and GRACE have been demonstrated in West Africa [11], the Nepal Himalayas [12] and southern Alaska [13]. Perhaps due to technical errors in GPS data processing, poorer agreement between GRACE and GPS has also been reported over Europe [14] and over Central America [15]. In addition, Wang *et al.* [16] used GPS measurements to separate the strong background signals of glacial isostatic adjustment (GIA) from GRACE estimates in North America and Scandinavia. Argus *et al.* [17] inverted the GPS observations of seasonal vertical motion to infer changes in equivalent water thickness in California by using GRACE estimates as a constraint of total water storage change.

**Figure 1.**
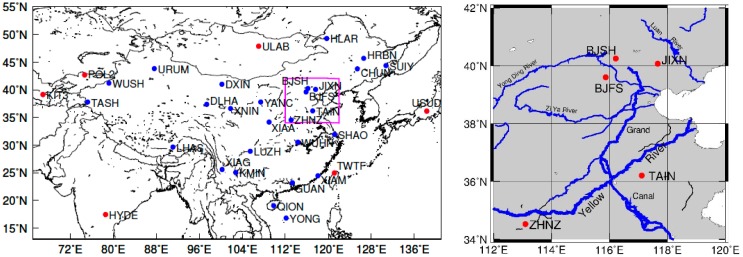
(Left) Distribution of continuous GPS stations (blue dots) and selected IGS stations (red dots) (**Right**); Enlarged view of the box area (**Left**) displayed with the Yellow River and its major tributaries in the study area.

However, relatively fewer studies on regional deformation due to hydrologic loading have been done in regions with much smaller seasonal amplitude, such as the North China Plain (Figure 1). The North China Plain is a region where the precipitation is low and ∼68% of agricultural irrigation comes from groundwater, and hence, its hydrologic signal is expected to be smaller than those of the aforementioned studies. The water usage for industrial and domestic purposes is continuously expanding [18–20]. Due to this usage, the groundwater depletion and the total water loss based on GRACE have been recently reported to be 2.26 ± 0.3 cm/year from 2003 to 2010 [21], and 1.3–2.4 cm/year from 2003 to 2006 for the North China Plain [22], respectively.

In this paper, we conduct a study using the latest GPS and GRACE data processing strategies to: (1) investigate the characteristics of seasonal mass changes and the corresponding secular vertical displacements in the North China Plain; (2) compare and cross-validate both the two geodetic observations; and (3) discuss and interpret the potential discrepancies between GRACE and GPS results in terms of spatial resolution and observation error sources.

## 2. Methods and Data Processing

### 2.1. Analysis of GPS Data

Twenty-seven continuous GPS sites in the Crustal Movement Observation Network of China (CMONOC) and six IGS sites (Figure 1) have been used to form a baseline network for GAMIT GPS solutions. All the GPS stations have been continuously observed for more than ten years. To keep the same data time span with GRACE data, the GPS data between January 2004 and December 2010 were used. GAMIT software was employed in “baseline mode” to process the GPS data, to obtain daily coordinates and covariance in the ITRF2008 reference frame, which corresponds to fixing the satellite orbits, and simultaneously estimating the site coordinates, tropospheric delay and the horizontal gradient parameters [23].

Solid-Earth tides [24], ocean tidal loading [25], and pole tides [26] have been corrected, whereas non-tidal loading was not corrected according to the 2010 IERS Conventions [27]. The impacts of second-order and third order ionospheric delay were also considered, in which the International Geomagnetic Reference Field 11 (IGRF 11) was selected to calculate the second-order ionospheric delay [28].

Gross error rejection was implemented to the baseline resolutions using GLOBK software and datum transformation was performed to obtain station coordinate time series with respect to the ITRF2008 reference frame [29]. In the datum transformation process, only six parameters (*i.e.*, three rotation and three translation parameters) were estimated in order to reduce the aliasing effects of the unmodeled surface mass loadings at seasonal scale [30].

Since this paper focuses on the seasonal variations of the surface deformation due to hydrologic loading, only data on Wednesdays during the period of January 2004 to December 2010 are utilized for the analysis. To avoid the observations of land surface subsidence due to sediment compaction in the aquifer caused by groundwater depletion, all selected GPS sites are located on bedrock (Table 1) which can be confirmed from CMONOC GPS site information published on its website [31].

### 2.2. Processing and Analysis of GRACE Data

#### 2.2.1. Post-Processing of GRACE Monthly Gravity Field Solutions

**Figure 2.**
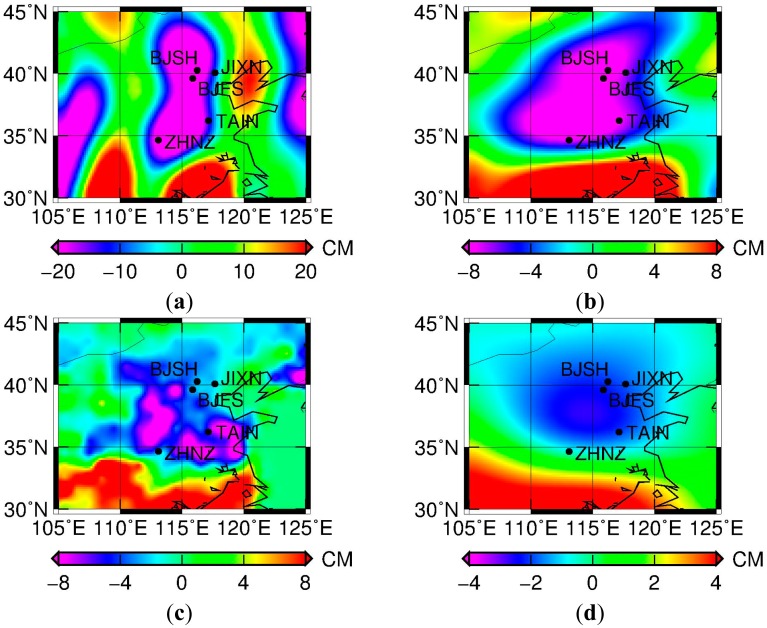
Total water storage (in terms of equivalent water height) changes derived by GRACE and GLDAS monthly data of July 2010 with respect to the mean field from 2004 to 2010. (**a**) Derived from GRACE without filtering; (**b**) Derived from GRACE using destriping and smoothing algorithms (*i.e.*, (55, 0) ↔ (20, 20), *k* = 20 decorrelation and *r* = 300 km Gaussian smoothing); (**c**) Derived from GLDAS without filtering; (**d**) Derived from GLDAS using destriping and smoothing algorithms (*i.e.*, (55, 0) ↔ (20, 20), *k* = 20 decorrelation and *r* = 300 km Gaussian smoothing). The black dots are locations of GPS sites. Note the different scale of the subfigures.

The monthly GRACE Level-2 Release-05 (RL05) solution GSM gravity data product, in the form of spherical harmonics coefficients (SHCs) provided from the University of Texas Center for Space Research (UTCSR), for the period between January 2004 and December 2010, up to degree 60, was used. Glacial Isostatic Adjustment (GIA) and an averaged mean gravity field model between 2004 and 2010 were first removed from the data product, in which the GIA model by Paulson *et al.* [32] was used. A global distribution of equivalent water height (EWH) change computed from unsmoothed GRACE SHCs is dominated by high-frequency errors. The most obvious characteristic is the presence of north-south stripes (Figure 2a), indicating spatially correlated error in the GRACE observations at higher frequency. This results from a combination of instrument, orbit and model errors causing an ill-conditioned gravity field inversion, manifesting the errors at geo-potential resonant orders [33]. Therefore, GRACE monthly temporal gravity field solutions have to be destriped or decorrelated, and spatially smoothed [34].

The effect of the decorrelation and spatial smoothing, shown in Figure 2a and b, are in the form of GRACE EWH changes, which can be computed using residual SHC data. As compared to Figure 2a, most stripes are removed in Figure 2b, resulting in a smoother monthly mass distribution map. However, decorrelation and spatial smoothing may distort the real signal and reduce the spatial resolution during the error reduction procedure. To illustrate this effect in a clear manner, the same decorrelation and smoothing process was applied to Global Land Data Assimilation System (GLDAS) NOAH hydrologic model data [35] (Figure 2d). Compared to the original GLDAS data (Figure 2c), the filtered result (Figure 2d) shows fewer details. In other words, the stronger the filtering is, the more details of the signal are lost.

**Figure 3.**
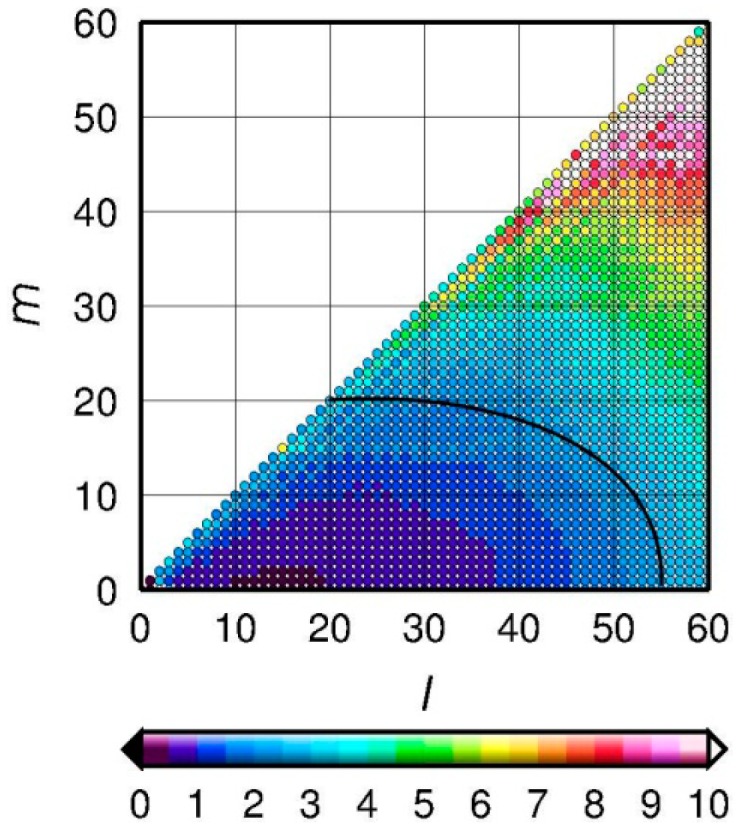
Error (scaled by ×10^−12^) in degree *l* and order *m* of residual spherical harmonic coefficients for empirically determining the boundary for keeping low degree of SHCs portion unchanged in the decorrelation processing (*i.e.*, the black curve).

Therefore, a compromise should be made among the short wavelength errors at higher degree, the signal distortion, and the spatial resolution for the decorrelation and spatial smoothing processes according to the SHC error characteristics. We adopt the destriping method of Duan *et al.* [36] which contains two steps: (1) the unchanged lower degree-order boundary of the SHCs are empirically defined using coordinate pairs (*l*_1_, *m*_1_) ↔ (*l*_2_, *m*_2_) as shown in the black curve of Figure 3, where *l* and *m* are the degree and the order, respectively. The purpose of keeping the lower degree-order portion of the SHCs unchanged in the destriping process is because it has lower uncertainty when compared to that of the higher degree-order portion (Figure 3) and (2) a moving window of width *k* is used to fit the correlated part, followed by subtracting this correlated part from SHCs. After several experiments and consideration of the aforementioned compromise, a boundary of keeping unchanged low degree-order of SHCs portion, defined by coordinate (*l*, *m*) end-point pairs (55, 0) ↔ (20, 20) with filtering window width *k* = 20, and *r* = 300 km for the decorrelation and Gaussian smoothing radius, respectively, were chosen to minimize the GRACE errors while keeping the optimal filtering.

#### 2.2.2. Vertical Surface Deformation due to Loading

Vertical surface deformation or displacement due to changing mass loading can be expressed in terms of residual spherical harmonics coefficients of gravity field and load Love number as follows [10,14,37]:

(1) $$dr\left(\theta ,\lambda \right)=R\sum_{l=1}^{\infty }\sum_{m=0}^{l}{\bar{P}}_{l,m}\left(\cos\theta \right)\cdot \left(\Delta C_{lm}\cos\left(m\lambda \right)+\Delta S_{lm}\sin\left(m\lambda \right)\right)\frac{h_{1}^{\prime }}{1+k_{1}^{\prime }}$$

where *dr*(θ,λ) is the displacement of the Earth surface in the radial direction; *R* is the average Earth radius; *P̄_l,m_* are fully normalized Legendre functions of degree *l* and order m; Δ*C_lm_* and Δ*S_lm_* represent the residual spherical harmonics coefficients of the destriped and smoothed gravity field from which the average gravity field between 2004 and 2010 have been removed; $h_{l}^{\prime }$ and $k_{l}^{\prime }$ are Load Love numbers at degree *l*; here, we adopt the load Love numbers from Guo *et al.* [38], which has used the PREM Earth model, improved the asymptotic expressions of load Love numbers and attained better results compared with Farrell [1], to transform these residual coefficients into vertical surface deformation estimates.

#### 2.2.3. AOD1B Effects and Geocenter Motion Correction

In order to consistently compare GPS and GRACE time series, we added back the de-aliasing atmospheric and non-tidal oceanic effects to the GRACE data, primarily because these effects cannot be easily removed from the GPS height time series.

Atmosphere and Ocean De-aliasing Level-1 B (AOD1B) product (GAC) is a product for adding back the atmospheric and non-tidal ocean loading effects. It accounts for the daily variability generated from the European Centre for Medium-Range Weather Forecasts surface atmospheric pressure and ocean mass as determined from a barotropic ocean model [39,40], in the form of change in gravity field (as expressed in the spherical harmonic coefficients (SHCs) up to degree and order 100).

**Figure 4.**
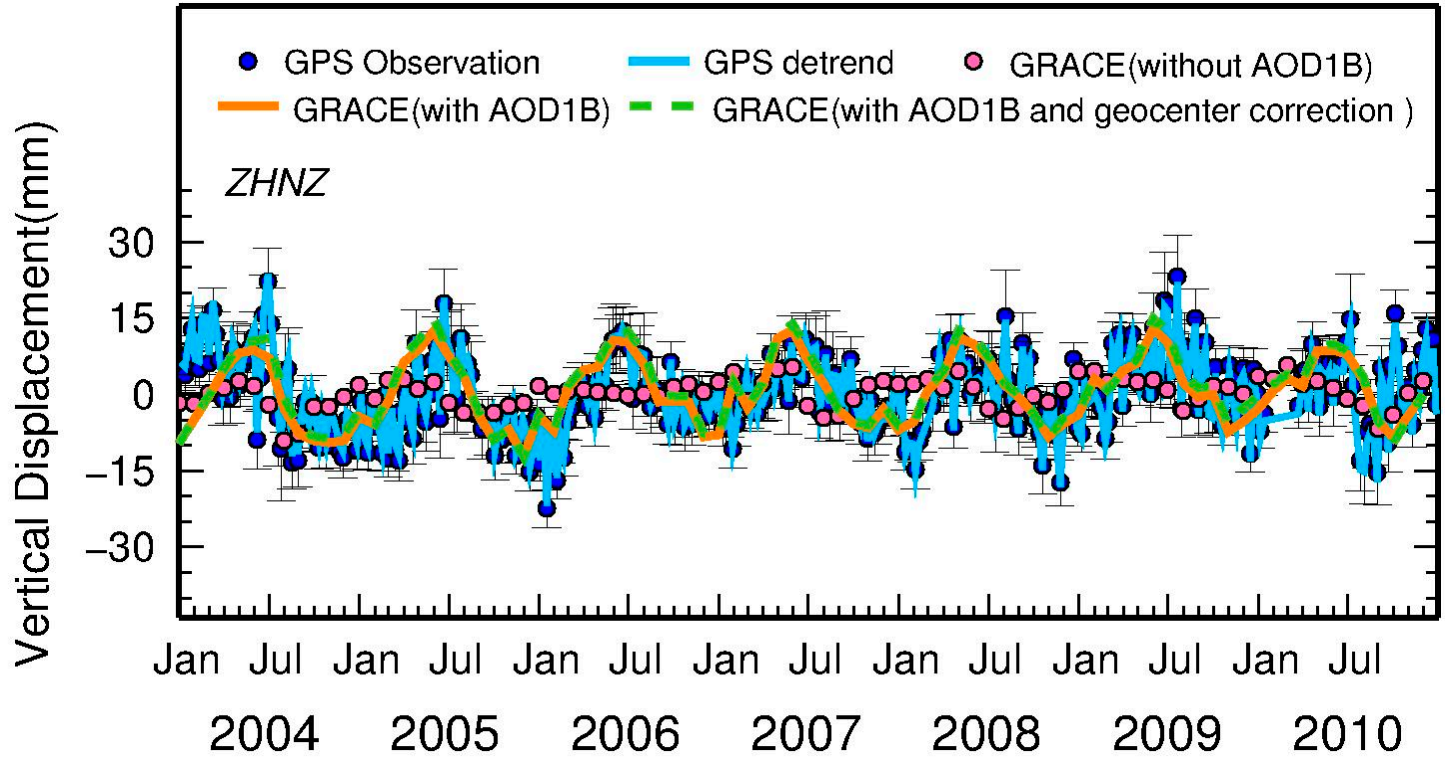
Vertical surface displacements (mm) derived from GPS observations and its detrended seasonal time series, and GRACE with or without AOD1B and geocenter correction, using ZHNZ GPS station as an example.

To demonstrate this effect, in Figure 4, the pink dots and orange line show GRACE-derived vertical displacements collocated at a GPS station (called ZHNZ) before and after adding back AOD1B product. It is apparent that the amplitude of GRACE solutions with AOD1B (the orange line) is larger than that without AOD1B (the pink dots), in which the former shows a good agreement with the detrended GPS variations (the cyan line) for both amplitude and phase. As a result, a consistent treatment of atmospheric and non-tidal ocean loading is necessary for GPS and GRACE solutions.

GRACE temporal gravity field has no geocenter motion contribution (*l* = 1), as GRACE is not sensitive to degree 1 signals. GRACE data are with respect to the center of mass (CM) of the Earth system, whereas detrended GPS data are with respect to the center of Figure (CF) of the Earth (e.g., International Terrestrial Reference Frame (ITRF)) [41,42]. Thus, we corrected the *l* = 1 term contribution to GRACE-derived mass variations, using the geocenter derived from satellite laser ranging (SLR) data, in order to be consistently comparable to the GPS time series. The relationship between geocenter data {Δ*Z_CM_*, Δ*X_CM_*, Δ*Y_CM_*} and degree 1 Stokes coefficients {Δ*_r_C*_1,0_, Δ*_r_C*_1,1_, Δ*_r_S*_1,1_} are shown in Equation (2) as follows:

(2) $$\left\{\Delta_{r}C_{1,0},\Delta_{r}C_{1,1},\Delta_{r}S_{1,1}\right\}=\frac{1}{\sqrt{3}R}\left\{\Delta Z_{CM},\Delta X_{CM},\Delta Y_{CM}\right\}$$

In this work, we use the geocenter data of UTCSR monthly geocenter RL-05 time series from SLR to obtain degree-1 Stokes coefficients, followed by the use of Equation (1) to get degree-1 contribution to vertical deformation, here the value of the degree 1 load Love number in the CF frame should be used [41].

To demonstrate the effect, the vertical displacements derived from GRACE before (green line) and after (orange line) geocenter correction is shown in Figure 4. The difference between them is not as apparent as the case with or without AOD1B. Though the difference is not readily apparent, the geocenter motion effect is substantial for the selected GPS stations in which the average effect can be more than 1 mm.

## 3. Results

### 3.1. Analysis of Vertical Surface Displacement

The vertical displacement seasonal variations (at annual and semi-annual periods) and the secular trend observed from GPS sites (*i.e.*, BJFS, BJSH, JIXN, TAIN and ZHNZ) and predicted from GRACE are compared (Table 2). This is achieved by a simultaneous fit for the mean, the trend, and the annual and semi-annual signals.

The GPS- and GRACE-derived trends show uplift of 0–2 mm/year for all GPS stations, which implies a continuous decrease in the water storage in the North China Plain. This result is consistent with the recent findings that the groundwater depletion and the total water loss based on GRACE is 2.26 ± 0.3 cm/year from 2003 to 2010 [21], and 1.3–2.4 cm/year for the period from 2003 to 2006 for the study region [22], respectively.

**Figure 5.**
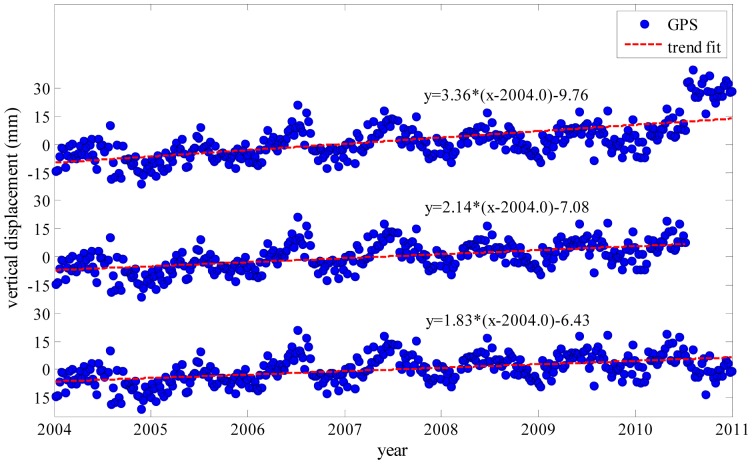
Estimated trend differences due to an offset occurred at the end of JIXN GPS time series near the end of 2010: (top) without applying the offset correction; (middle) deleting the end of data time series where the offset occurs; (bottom) applying the offset correction to the end of data time series.

From Table 2, it is clear that the GPS-derived trend is a bit larger than that derived from GRACE, especially for GPS stations BJFS and JIXN. This should be mainly due to the higher spatial resolution of GPS observations [17,43] when compared to that of GRACE estimates. In addition, GRACE solutions are corrected for GIA while GPS ones are not, which is about 0.15–0.20 ± 0.04–0.06 mm/year in the North China Plain [44]. The removal of GPS antenna offset may also introduce errors. Taking station JIXN as an example, the original time series of JIXN displays an apparent offset at the end of June 2010 (Figure 5), which is due to the upgrade of GPS antenna with different heights on 22 June 2010. Therefore, the removal of this offset may potentially introduce an error. Besides, the GPS-derived trends can be caused by hydrologic process, thermal expansion of bedrock due to temperature, to mention a few [42], while GRACE results mainly reflect the hydrologic process after correcting for GIA effects with a model [45].

**Figure 6.**
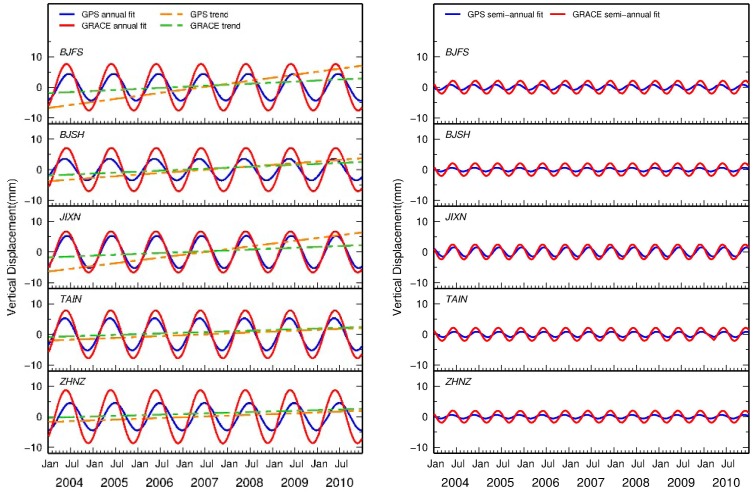
GPS- and GRACE-derived trends, annual and semi-annual signals between 2004 and 2010.

Seasonal variations of vertical surface displacement are significant in both GPS and GRACE solutions (Figure 6). For all the selected GPS sites, the annual component is more dominant than the semi-annual one. The peak-to-peak annual amplitude is 3.8–5.6 mm and 6.7–8.7 mm for the GPS and GRACE solutions, respectively, while the semi-annual amplitude is about 1 mm and 2 mm, respectively. Compared to GPS solutions, the spatial coherence of seasonal amplitudes (the red line) and phases from GRACE solution are more apparent because spatial smoothing process has been applied to the GRACE estimates (*i.e.*, >300 km). The remarkable seasonal variations of GRACE solution time series reflect the seasonal hydrologic process. For these selected GPS sites, water volume content stored in winter is larger than that in summer. This is because underground water is largely pumped for agricultural usage in late spring and summer, whereas the water due to raining in autumn and winter remains in storage till next spring, apart from evaporation and runoff.

### 3.2. Quantitative Comparison of Seasonal Signals

GPS and GRACE solutions for all the selected GPS stations exhibit similar seasonal patterns for both amplitude and phase (Figure 6). To quantitatively evaluate the consistency of seasonal variation between GPS and GRACE, the relative correlation coefficients of seasonal variation between GPS and GRACE are computed. All the selected sites show high correlation (85.0%–98.5%) (Table 3), indicating that the seasonal variations might come from the same geophysical process. GRACE-derived seasonal deformation is also subtracted from GPS detrended height time series to compute the variance reduction ratio in terms of weighted root-mean-square (WRMS) (Table 3) based on the following equation [14].

(3) $$WRMS_{\text{reduction}}\left(\%\right)=\frac{WRMS_{GPS}-WRMS_{\text{GPS}-\text{GRACE}}}{WRMS_{GPS}}$$

The WRMS residual reduction ratio for all the stations ranged from 2.3% to 19.2%, which is better than that observed in Europe [14]. However, our resulting variance reduction is smaller when compared to that in Nepal, the Himalayas with an overall reduction of 45.5% [12], because the seasonal hydrologic process is more apparent, whose peak-to-peak seasonal amplitude can be more than two centimeters and more than twice times of the hydrologic signal in our study region. Besides, it is clear from the time series that the amplitude of seasonal signal is not constant. Davis *et al.* [46] discovered that the amplitude of seasonal (annual and semi-annual, *etc.*) signals varies with time in geodetic time series (both GRACE and GPS). As a result, the removal of traditional seasonal signals (assuming constant amplitude) from geodetic time series leaves a strong stochastic seasonal component. This implies data fitting processing strategies have a significant impact on the resulting seasonal estimates.

### 3.3. Interpretation of Discrepancy and Error Sources

From Table 2, the uncertainty of the GPS annual amplitude is about 0.3–0.5 mm, which is relatively high when compared to the estimated seasonal signals of 3.8–5.6 mm. This is because of the inherent weakness of the GPS height time series caused by observation geometry. For GRACE solutions, the annual amplitude and uncertainty is about 6.7–8.7 mm and ±0.05–0.06 mm respectively, which is far smaller when compared to the estimated amplitude.

**Figure 7.**
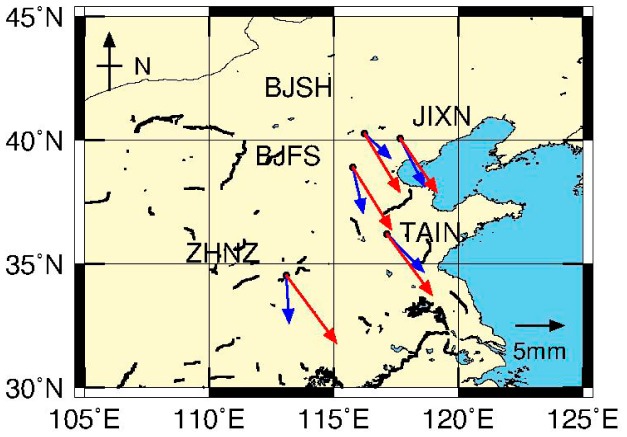
Comparison of annual amplitudes and initial phases between GPS (blue) and GRACE (red). The amplitude *A* and initial phase *f* are defined as *A*sin[*w*(*t* − *t*_0_) + *f*], where *t*_0_ is 2004.0 and *f* is the angular initial phase lag in degrees. The blue and red vectors represent the amplitudes and initial phases derived from GPS and GRACE. The initial phases are counterclockwise from the east.

To discuss the discrepancy between GPS and GRACE derived seasonal signals and the potential error sources, the estimated annual amplitudes and initial phases derived from GPS (blue vector) and GRACE (red vector) are shown in Figure 7. We observe that the amplitude of GRACE is relatively larger than that of GPS. Note that GRACE-derived seasonal signals represent large-scale (regional) averaged quantities, whereas the GPS seasonal signals are the loading response at a single point. Their difference should be mainly attributable to signal leakage when smoothing was performed for the GRACE solutions, where all the selected GPS sites located near but not on the main areas of load change. As displayed in Figure 2b,c, the leakage effect for the GRACE solutions after smoothing is too large when compared to GLDAS hydrologic model data that should have a higher resolution. On the other hand, there is a substantial loading effect on the terrestrial reference frame alignment [47,48]. An overall improvement of ∼0.2 mm (in terms of transformation fitting) can be made at 70%–80% success rate in a regional network [49]. The phase of GRACE solutions also exhibits an apparent difference from that of GPS. There are two reasons explaining this fact. One is that the Gaussian filtering process introduces phase changes, possibly because of asymmetric spectral leakage errors from surrounding basins, which might shift the annual phases by up to 10 degrees [50]. Another plausible reason is different temporal sampling rate of GPS when compared to that of GRACE. The GPS surface deformations due to hydrologic loading can be observed right after mass redistribution, while GRACE reflects this variation at a monthly scale. This geophysical process explains the difference of initial phase.

For GPS, data processing strategies would ultimately affect the annual signal of height residuals, which might be another reason for the discrepancy. For instance, Penna *et al.* [51] found that the ocean model errors would cause more than 1 mm uncertainty in the estimated annual amplitude of the vertical surface displacement. Horwath *et al.* [52] found that solar radiation pressure and Earth albedo model might generate the annual signal. The selection of mapping function in the troposphere model would affect the determination of the seasonal signals from height residuals. Yan *et al.* [53] considered that the temperature might be a non-negligible factor for annual part of GPS vertical time series. They found that the annual amplitude of vertical surface displacement caused by GPS station bedrock temperature variation in China could be up to 1 mm. Further discrepancy causes include the impacts on frame transformation parameters [47–49] and non-tidal ocean loading [1] effects on the GPS height time series and the smoothing impact of GRACE solutions.

## 4. Discussion and Conclusions

GRACE-derived vertical displacements due to seasonal hydrologic loading showed high correlation (85.0%–98.5%) with GPS observed seasonal position variations. The groundwater has been depleted in the North China Plain, which causes two geophysical processes. One is the land subsidence due to compaction of sediments in the aquifer, while another is the uplift due to the reduction of the surface loading. The first geophysical process likely affects only sites in soil or sediments but not bedrock sites. As all the GPS stations selected here are installed on bedrock, we conclude that the main cause of seasonal position variation in the North China Plain should be due to hydrologic mass loading. This seasonal hydrologic loading deformation reflects the groundwater pumping activities for agriculture irrigation in spring, leading to less water storage in summer than in winter.

In addition, we found that WRMS reduction ratio after removing the GRACE-derived elastic deformation from the coordinate time series ranges from 2.3% to 19.2%. This might be explained by the smaller hydrologic signal and the higher frequency fluctuation caused by non-tidal ocean loading effects. These effects display an 0.2–3.7 mm RMS scatter in the height residuals [54,55], in which the effects at stations located close to semi-enclosed bays or seas are larger than other stations. Moreover, Nordman *et al.* [2] found that removing the computed non-tidal ocean loading from the GPS time series reduced the standard deviation for the selected GPS sites close to the Baltic Sea, with the reduction ranging from 23% to 43%. The latitudes of GPS sites range between N34° and N41° are near the Bohai Sea. The non-tidal ocean loading, certainly affect the GPS time series in this region. Besides, some kind of noises in the GPS time series may be associated with the ignorance of the stochastic seasonal signal component, which has been discussed in Section 4.

The results presented in our study demonstrate that the seasonal height variation from GPS sites in the North China Plain agrees well with the seasonal signal predicted by the terrestrial water storage loading signal estimated from GRACE time-variable monthly gravity field. Further studies involve consideration of the spatial smoothing impact of GRACE solutions, the non-tidal ocean effect on the GPS height time series, as well as the stochastic seasonal signal component in geodetic time series [46], which might likely be the cause of the low WRMS reduction ratio.

## Figures and Tables

**Table 1.** Specific information of selected GPS sites.

**Site Code**	**Longitude (°)**	**Latitude (°)**	**Site Geologic Characteristic**	**Site Start Date**
BJFS	E115.88°	N39.60°	Shale, dry rock, and little slate	1995–2009
BJSH	E116.22°	N40.25°	Limestone	1998–2009
JIXN	E117.67°	N40.07°	Sinian limestone	1998–2007
TAIN	E117.12°	N36.20°	Archean granite schist	1998–2007
ZHNZ	E113.10°	N34.52°	Triassic continental sandstone	1998–2004

**Table 2.** Seasonal (*i.e.*, annual and semi-annual) amplitudes and phases, trend fit of vertical displacements derived by GRACE and GPS for selected GPS stations, and their corresponding goodness-of-fit value in terms of Root-Mean Square Error (RMSE). Note that the semi-annual amplitudes and phases are listed in the corresponding brackets.

**GPS Station Code**	**Annual and (semi-annual) Amplitude (mm)**		**Annual and (semi-annual) Phase (days)**		**Trend Rate (mm/year)**		**RMSE (mm)**	
	**GRACE**	**GPS**	**GRACE**	**GPS**	**GRACE**	**GPS**	**GRACE**	**GPS**
BJFS	7.63 ± 0.06 (2.08 ± 0.04)	4.91 ± 0.41 (0.86 ± 0.50)	306.67 ± 28.21 (82.83 ± 17.54)	287.94 ± 15.10 (99.07 ± 15.68)	0.69 ± 0.06	1.98 ± 0.11	2.55	5.76
BJSH	7.10 ± 0.05 (1.92 ± 0.04)	3.82 ± 0.27 (0.60 ± 0.23)	306.04 ± 29.50 (84.76 ± 19.75)	321.56 ± 17.42 (90.36 ± 16.85)	0.64 ± 0.06	1.08 ± 0.04	2.51	5.34
JIXN	6.74 ± 0.05 (2.02 ± 0.04)	5.55 ± 0.39 (1.43 ± 0.28)	309.21 ± 27.91 (88.65 ± 30.53)	301.92 ± 18.01 (85.58 ± 17.42)	0.57 ± 0.06	1.83 ± 0.03	2.56	5.36
TAIN	7.84 ± 0.06 (2.39 ± 0.04)	5.49 ± 0.50 (0.88 ± 0.42)	311.39 ± 23.72 (89.29 ± 25.64)	319.10 ± 16.85 (54.92 ± 15.10)	0.47 ± 0.06	0.62 ± 0.04	2.78	5.46
ZHNZ	8.71 ± 0.06 (2.13 ± 0.04)	5.03 ± 0.34 (0.82 ± 0.38)	311.34 ± 25.13 (79.01 ± 10.81)	278.48 ± 16.26 (70.60 ± 20.91)	0.42 ± 0.06	0.60 ± 0.03	2.84	6.51

**Table 3.** Correlation between GPS and GRACE derived seasonal variations and WRMS reduction ratio of all selected GPS sites after removing GRACE-derived elastic deformation from GPS time series.

	**BJFS**	**BJSH**	**JIXN**	**TAIN**	**ZHNZ**
Correlation (%)	93.4	96.0	97.5	98.5	85.0
WRMS reduction (%)	13.6	2.3	19.2	5.6	6.8
